# New Appliances and Things Medical

**Published:** 1904-05-07

**Authors:** 


### NEW APPLIANCES AND THINGS MEDICAL.

[We shall be glad to receive at our Office, 28 & 29 Southampton Street, Strand, London, W.C., from the manufacturers, specimens of all new preparations and appliances which may be brought out from time to time.]

### WAMPOLE'S PERFECTED AND TASTELESS PREPARATION OF THE EXTRACT OF COD-LIVER OIL.

(Henry K. Wampole and Co., Manufacturing Chemists, Philadelphia, U.S.A. London Agents: Francis Newbery and Sons, Charterhouse Square, E.C.)

This preparation is a complex mixture, having no odour or taste of cod-liver oil, and containing only the aromatic principles of the latter, which most palates would regard as far from unpleasant. Its essential feature is said to be that it includes the alkaloids of cod-liver oil, while the fatty constituents of the oil are entirely eliminated.

Seeing that it is still undecided to what particular constituent the efficacy of cod-liver oil is due, there will probably occur to most minds a doubt whether, in removing the oil, the active principle also may not have been eliminated. This is a question which can only be cleared up by extensive clinical and experimental observation. In the meanwhile, therefore, we cannot undertake to recommend this preparation as a substitute for cod-liver oil simply on the ground of its being not so unpleasant to take. As the mixture contains extract of malt and compound syrup of hypophosphites, in addition to the so-called extract of cod-liver oil, it may be of use where extract of malt and hypophosphites are indicated. We cannot, however, but think that the name of the preparation is an unfortunate one, insomuch as it might lead people to believe that the mixture depends for its efficacy upon cod-liver oil, which of course it does not.

